# Supplementary figures and images for: CTLA-4Ig Improves Hyperalgesia in a Mouse Model of Osteoporosis
Source: Int J Mol Sci. 2020 Dec 13;21(24):9479. doi: 10.3390/ijms21249479 (PMC7763121; doi:10.3390/ijms21249479)

## Supplementary Materials

### S1: mRNA levels of TNF $\alpha$

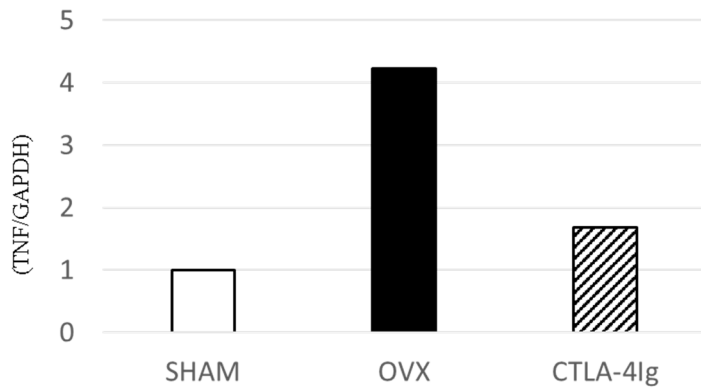

### S2: mRNA levels of SOST

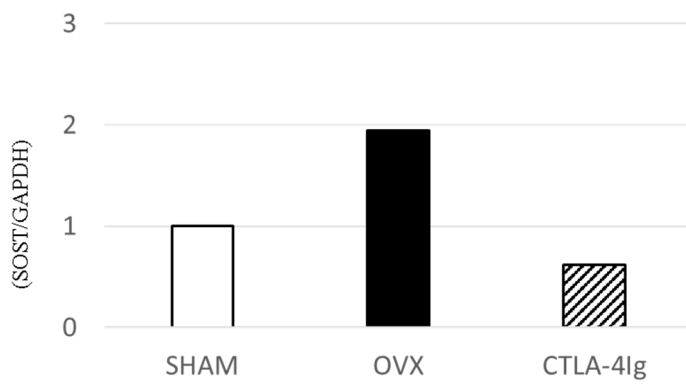

### S3: mRNA levels of Wnt-10b

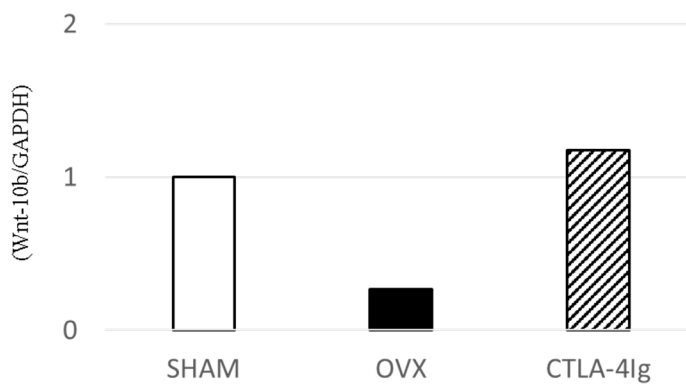

Supplement: Supplementary file 1 [file ijms-21-09479-s001.pdf]
